# The influence of bicarbonate concentration and ionic strength on peroxide speciation and overall reactivity towards UO_2_†

**DOI:** 10.1039/d4ra02281e

**Published:** 2024-05-20

**Authors:** Daniel Olsson, Hazal Aydogan, Mats Jonsson

**Affiliations:** a Department of Chemistry, KTH Royal Institute of Technology Stockholm SE-100 44 Sweden daniols@kth.se

## Abstract

H_2_O_2_ produced from water radiolysis is expected to play a significant role in radiation induced oxidative dissolution of spent nuclear fuel under the anoxic conditions of a deep geological repository if the safety-barriers fail and ground water reaches the fuel. It was recently found that the coordination chemistry between U(vi), HCO_3_^2−^ and H_2_O_2_ can significantly suppress H_2_O_2_ induced dissolution of UO_2_ in 10 mM bicarbonate. This was attributed to the much lower reactivity of the U(vi)O_2_^2+^-coordinated O_2_^2−^ as compared to free H_2_O_2_. We have extended the study to lower bicarbonate concentrations and explored the impact of ionic strength to elucidate the rationale for the low reactivity of complexed H_2_O_2_. The experimental results clearly show that dissolution of U(vi) becomes suppressed at [HCO_3_^−^] < 10 mM. Furthermore, we found that the reactivity of the peroxide in solutions containing U(vi) becomes increasingly more suppressed at lower carbonate concentration. The suppression is not influenced by the ionic strength, which implies that the low reactivity of O_2_^2−^ in ternary uranyl-peroxo-carbonato complexes is not caused by electrostatic repulsion between the negatively charged complex and the negatively charged UO_2_-surface as we previously hypothesized. Instead, the suppressed reactivity is suggested to be attributed to inherently higher stability of the peroxide functionality as a ligand to UO_2_^2+^ compared to as free H_2_O_2_.

## Abstract 2

Coordination of H_2_O_2_ by UO_2_^2+^ suppresses reaction between H_2_O_2_ and UO_2_ in bicarbonate solutions. The suppression is greater at lower [CO_3_^2−^] due to higher fractions of (UO_2_)_*x*_(O_2_)_*y*_(CO_3_)_*z*_^2*x*−2*y*−2*z*^.

## Graphical abstract

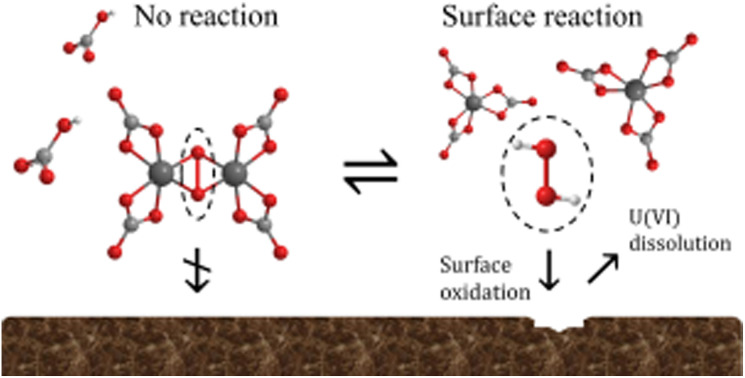

## 1. Introduction

Uranium dioxide (UO_2_) is used as the nuclear fuel in most commercial Light Water Reactors (LWR). After use in the reactor, the fuel matrix still consists of ∼95 percent UO_2_ and a small percentage of fission products and heavier actinides responsible for the increased radioactivity that persists long after the fuel has been removed from the reactor.^1^ Confinement of the spent fuel in the geosphere has been internationally accepted as the safest option to handle nuclear waste.^1*a*,2^ This solution is not without challenges as both natural and engineered barriers will have to withstand corrosion and mechanical stress for at least 100 000 years to be considered safe.^3^ If the barriers are breached, the nuclear fuel will be in contact with groundwater.

Predictive modeling of the release of long-lived and radiotoxic actinides and fission products upon groundwater intrusion largely relies on accurate prediction of the dissolution rate of the fuel matrix (mainly UO_2_).^4^ UO_2_ has low solubility under the reducing conditions expected at potential repository sites.^5^ However, oxidation of U(iv) occurs as a result of water radiolysis as the groundwater is exposed to ionizing radiation leading to the formation of strong oxidants (as well as strong reductants).^6^ It has been shown that U(vi) dissolution under typical repository conditions can mainly be attributed to oxidation induced by the aqueous radiolysis product H_2_O_2_.^7^ The solubility of U(vi) increases significantly by the presence of bicarbonate (expected in concentrations 1–10 mM in groundwater).^8^

The reaction between H_2_O_2_ and UO_2_ is known to occur as a competition between oxidation of UO_2_ (leading to oxidative dissolution) and UO_2_ catalyzed decomposition of H_2_O_2_ leading to the formation of oxygen and water but leaving the UO_2_ surface unaffected.^9^ It was previously found that the rate of the reaction between H_2_O_2_ and UO_2_, but not the oxidative efficiency (the dissolution yield) is influenced by the U(vi) concentration in 10 mM bicarbonate.^10^ This was attributed to varying fractions of stable uranyl-peroxo-carbonato complexes limiting the amount of reactive H_2_O_2_ available to the UO_2_ surface. In such a system the overall reaction scheme can be described as:

1 *p*UO_2_^2+^ + *q*H_2_O_2_ + *r*CO_3_^2−^ ⇆ [(UO_2_)_*p*_(O_2_)_*q*_(CO_3_)_*r*_]^2*p*−2*q*−2*r*+2*q*H+^ (ref. **11**)

2
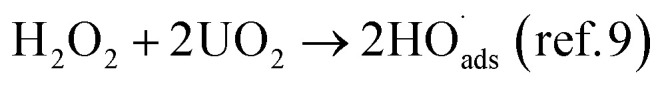
3
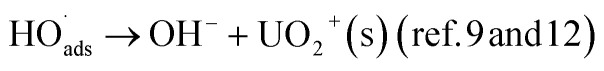
4
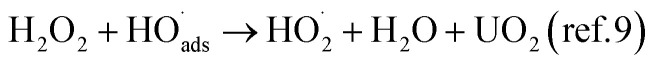
5
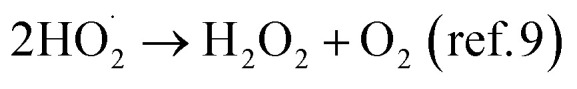


Both H_2_O_2_-induced oxidation of UO_2_ (reaction (3)) and UO_2_-catalyzed decomposition of H_2_O_2_ (reactions (4) and (5)) have the surface bound hydroxyl radical as a common intermediate. The U(v) produced in reaction (3) is further oxidized into U(vi) in a subsequent step. Direct oxidation of U(v) by OH˙ and disproportionation of two U(v) into U(iv) and U(vi) have been proposed as two possible mechanisms.^12,13^ Dissolution of U(vi) follows the second oxidation step as a result of complexation reaction(s) between U(vi) and carbonate:

6 UO_2_^2+^_(s)_ + *n*CO_3_^2−^ → UO_2_(CO_3_)_*n*_^2−2*n*^_(aq)_ (ref. **1*b***)

The equilibria described by reaction (1) indicates that dissolved U(vi) could theoretically suppress the dissolution rate (reaction (6)) by binding carbonate ligands as well as the rate of oxidation (reaction (3)) by binding peroxo-ligands. The affinity of U(vi) towards peroxo- and carbonato ligands follows the order CO_3_^2−^ < O_2_^2−^.^14^ Groundwaters present at different potential repository sites can vary in terms of composition. As mentioned above, the concentration of HCO_3_^−^/CO_3_^2−^ can vary by one order of magnitude. The ionic strength also varies between sites. In our previous work, we explored the impact of peroxide speciation on the overall peroxide reactivity at 10 mM HCO_3_^−^ and constant ionic strength (mainly determined by NaHCO_3_). Hence, the impact of HCO_3_^−^/CO_3_^2−^ concentration as well as ionic strength on this system must be explored before peroxide speciation can be correctly accounted for in safety assessments of geological repositories for spent nuclear fuel.

In this work the impact of uranyl-peroxo-carbonato speciation on the overall kinetics of H_2_O_2_ induced dissolution of hyper-stoichiometric UO_2_ has been studied at various carbonate concentrations within the range 1–10 mM. In addition, the ionic strength dependence on the peroxide reactivity was studied as a means of testing if the lower reactivity of the peroxo-ligands in the complexes is due to electrostatic repulsion between the negatively charged U(vi)-peroxo-carbonato complexes and the negatively charged UO_2_ surface as was previously hypothesized.^10,15^

## 2. Materials and methods

The chemicals used throughout the experiments were of reagent grade or higher. Stock solutions of UO_2_(NO_3_)_2_ × 6H_2_O (Westinghouse AB), H_2_O_2_, KI, Arsenazo-III, NaClO_4_ × H_2_O (Sigma Aldrich) and NaHCO_3_ (Merck) where prepared using ultra-pure water (18.2 MΩ cm, Merck MilliQ).

The UO_2_ powder (Westinghouse AB) used throughout these experiments has the specific surface area (BET) 5.4 ± 0.2 m^2^ g^−1^ and oxidation state UO_2.34_, as determined in a previous work.^9*b*^ The powder was washed prior to exposure to remove U(vi) (*i.e.*, pre-oxidized UO_2_). The washing procedure has been described in detail elsewhere.^10^

The speciation was varied by an initial addition of uranyl nitrate. Speciation was simulated in SPANA,^16^ using stability constants published by Zanonato *et al.* and ionic strength correction based on the Specific Ion-Interaction Theory (SIT) model.^11,17^ For convenience, the stability constants used for our speciation calculations are listed in Table 1.

**Table 1:** Stability constants used for speciation calculations

Species	Log_10_ *β*^11^
HO_2_^−^	−11.29
OH^−^	−13.72
HCO_3_^−^	9.69
H_2_CO_3_(g)	15.60
(UO_2_)_2_(OH)_2_^2+^	−6.07
UO_2_(OH)_3_^−^	−19.69
UO_2_(CO_3_)_3_^4−^	21.76
UO_2_(CO_3_)_2_^2−^	14.93
UO_2_CO_3_	8.57
(UO_2_)_3_(CO_3_)_6_^6−^	53.82
UO_2_(O_2_)(OH)^−^	−14.16
(UO_2_)_2_(O_2_)_2_(OH)^−^	−15.82
(UO_2_)(O_2_)(CO_3_)_2_^4−^	4.37
UO_2_(O_2_)(CO_3_)^2−^	1.47
(UO_2_)_2_(O_2_)_2_(CO_3_)^2−^	1.98
(UO_2_)_2_(O_2_)(CO_3_)_2_^2−^	18.31
(UO_2_)_2_(O_2_)(CO_3_)_4_^6−^	27.4
(UO_2_)_2_(CO_3_)(OH)^3−^	−1.89
UO_2_(O_2_)(OH)^−^	−2.67
(UO_2_)_2_(O_2_)(OH)^−^	7.16

The interactions between UO_2_^2+^ and NO_3_^−^ were not included in the speciation calculations as they are too weak to significantly affect the speciation under the conditions used in this study. The apparent equilibrium constants (*β*) found in literature for the UO_2_NO_3_^+^ complex at 25 °C are within the range 0.15 to 0.65,^18^ considerably weaker interactions than for the dominant uranyl-carbonato- and uranyl-peroxo-carbonato-complexes (see Table 1).

The concentration of U(vi) was measured by the absorbance of U(vi)-(1,8-dihydroxynaphthalene-3,6-disulphonic acid-2,7-bis[(azo-2)-phenylarsonic acid]) complex at 653 nm (Arsenazo III method)^19^ using calibrations determined through titration (available as ESI† to our previous work^10^). The total peroxide concentration (free H_2_O_2_ and U(vi)-coordinated O_2_^2−^) was measured indirectly by fully converting the peroxide into I_3_^−^ in the presence of a molybdenum catalyst (see Ghormley triiodide method^20^). I_3_^−^ strongly absorbs light at 360 nm where an overlap with the absorbance of U(vi) is avoided. The extinction coefficient for I_3_^−^ at 360 nm is 2.16 × 10^4^ dm^3^ mol^−1^ cm^−1^. Control experiments have shown that the results of the methods used to quantify uranium in solution Taand total peroxide in solution are not affected by speciation within the variation in this work.^21^

The pH was allowed to vary but was measured at several points during each experiment using a Thermo Scientific™ Orion Star™ A111 Benchtop pH Meter. Observed changes in pH were accounted for when performing speciation calculations. pH data for all exposures are included as ESI (Tables S1–S3).†

No blanks were used in this study to control for peroxide decomposition in the aqueous phase. As demonstrated in our previous work,^21^ decomposition in the aqueous phase has been found to be orders of magnitude slower than the decomposition of H_2_O_2_ on UO_2_ under conditions similar to those studied in this work.

## 3. Results

**Fig. 1:**
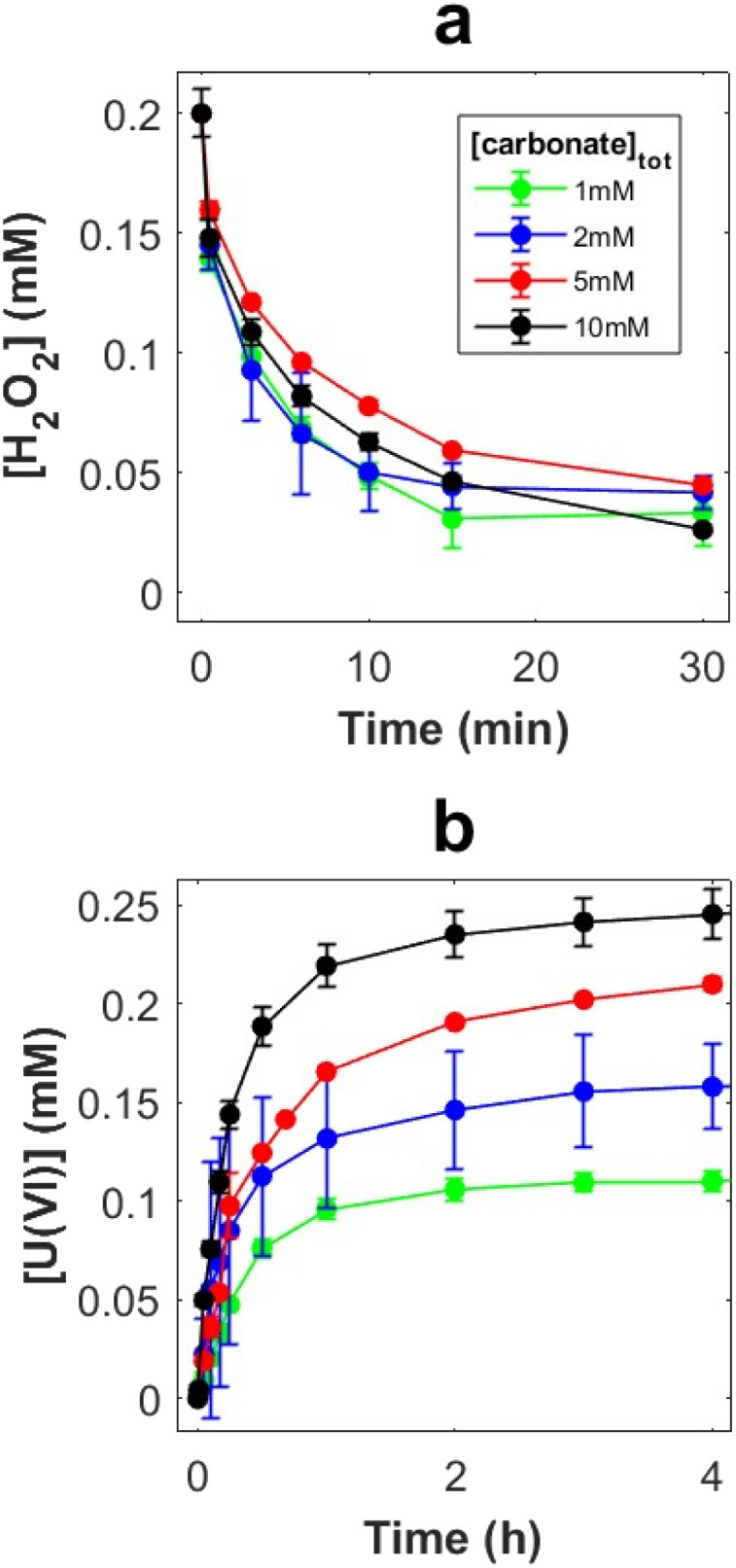
Peroxide concentration (a) and U(vi) concentration (b) as a function of exposure time in 30 ml bicarbonate solutions with 50 mg UO_2_ powder (SA/V = 9000 m^−1^).

A series of experiments were performed using an initial H_2_O_2_ concentration of 0.2 mM and bicarbonate concentrations of 1, 2, 5 and 10 mM in 30 ml solutions containing 50 mg UO_2_ powder. Total peroxide (H_2_O_2_ + peroxo-ligands) and U(vi) (UO_2_^2+^ + uranyl coordination centers) concentrations as function of time are presented in Fig. 1a and b, respectively.

Error bars represents standard deviations based on three replicate exposures. Note that while all other aliquot samples where both filtered and centrifugated, samples from the exposures using 2 mM bicarbonate were only filtered, resulting in larger uncertainty (likely caused by small UO_2_ particles remaining in the sample).

**Fig. 2:**
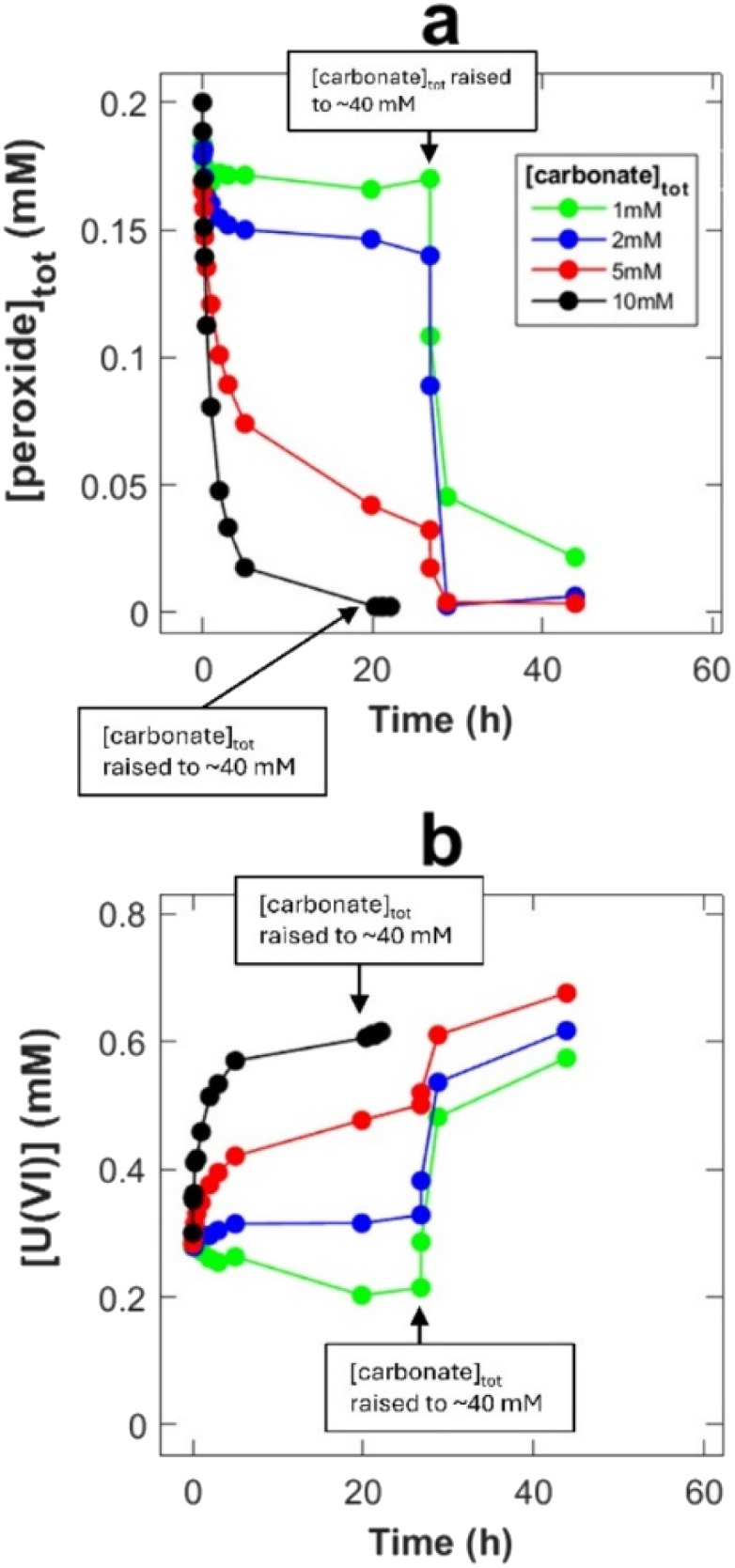
Peroxide concentration (a) and U(vi) concentration (b) as a function of exposure time in 20 ml solutions containing 1–10 mM bicarbonate, 0.2 mM initial peroxide and 0.3 mM initial U(vi), with 30 mg UO_2_ powder suspension (SA/V = 8100 m^−1^).

While the rate of peroxide consumption (decomposition *via* reactions (3) and (4)) was found to be similar at all four carbonate concentrations (Fig. 1a), there is a clear trend in the amount of dissolved U(vi), where the rate of dissolution as well as the final concentration (after 5 h) increases with increasing carbonate concentration. The ratio between dissolved UO_2_^2+^ and carbonate is probably crucial here. This possibility was further explored by adding 0.3 mM U(vi) prior to the experiment. In this series of experiments, the initial bicarbonate concentrations were the same as in the experiments without initially added U(vi) but towards the end of each exposure the concentration was increased to ∼40 mM. The late addition of carbonate was done to elucidate the reasons behind the limitation in uranium dissolution. The resulting peroxide concentrations and U(vi) concentrations as a function of time are presented in Fig. 2a and b, respectively.

**Fig. 3:**
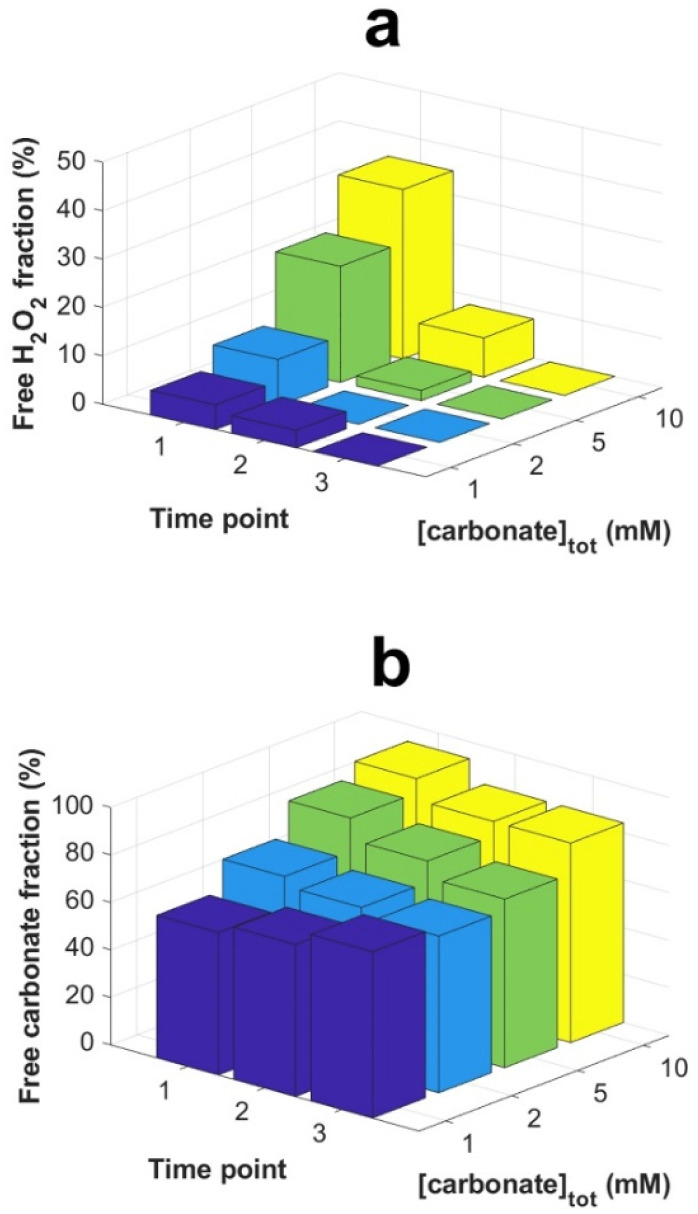
Calculated fractions of free H_2_O_2_ (a) and free carbonate species (HCO_3_^−^ + CO_3_^2−^) (b) as functions of time evolution and total carbonate concentration. Time points; 1 = start of reaction, 2 = after initial drop (5 h) and 3 = prior to bicarbonate addition (20 or 26 h), as indicated in Fig. 2.

**Fig. 4:**
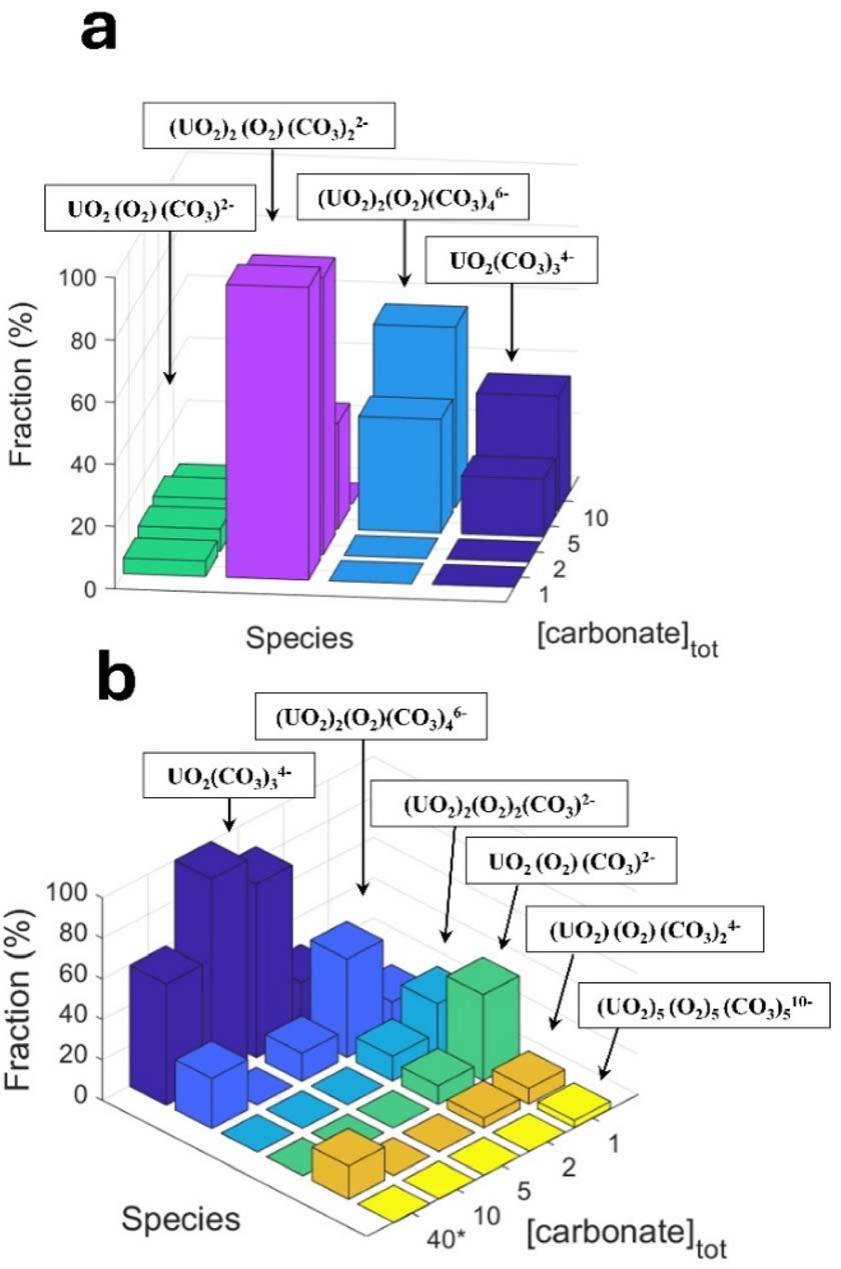
Calculated fractions of uranyl species as functions of total carbonate concentration at (a) the start of the exposures and (b) the time of NaHCO_3_ addition. *The 40 mM bicarbonate speciation was calculated for the 1 mM bicarbonate system after raising the total carbonate concentration.

As can be seen, the carbonate concentration dependence is even more pronounced in the systems initially containing 0.3 mM U(vi). In this case, also the peroxide consumption kinetics is significantly affected. Note that a lower SA/V was used when compared to the uranyl free systems in Fig. 1, which is expected to reduce the rate of a given surface reaction by 11 percent. To allow further analysis of the system, speciation calculations were performed. The calculated free peroxide (H_2_O_2_ + HO_2_^−^) – and free carbonate (HCO_3_^−^ + CO_3_^2−^) – as functions of time evolution and total carbonate concentration are presented in Fig. 3a and b respectively. The uranyl speciation as a function of total carbonate concentration at the start of the exposures and at the time of the carbonate additions are presented in Fig. 4a and b respectively. The speciation calculations were based on the data presented in Fig. 2a and b and the measured pH (see ESI, Fig. S2†).

Based on the calculated fractions of free H_2_O_2_ (Fig. 3a) and our previous conclusion that coordinated peroxides (O_2_^2−^ ligands) are passive towards the UO_2_ surface,^10^ kinetic suppression due to O_2_^2−^ coordination is expected to have a greater effect at lower bicarbonate concentrations, as a result of less competition from UO_2_(CO_3_)_3_^4−^ in favor of uranyl-peroxo-carbonato complexes (see Fig. 4a). The suppressing effect is also expected to increase with the progression of time due to the increase in uranyl concentration from oxidative dissolution of the UO_2_ powder. These predictions are in line with the observed trends for the peroxide concentrations as functions of time (Fig. 2a). Raising the total carbonate concentration from 1 to 40 mM is expected to increase the fraction of free H_2_O_2_ to ∼70 percent due to the formation of UO_2_(CO_3_)_3_^4−^ at the expense of uranyl-peroxo-carbonato complexes, while a small fraction of ternary complexes (∼20 and 10 percent respectively) is expected to exist in the form of (UO_2_)_2_(O_2_)(CO_3_)_4_^6−^ and (UO_2_)(O_2_)(CO_3_)_2_^4−^ as seen in Fig. 4b. The high increase in the fraction of free H_2_O_2_ at the moment of carbonate addition explains the rapid increase in reactivity towards the UO_2_ powder which is observed as the carbonate concentrations are raised.

Another possibility is that the suppression of the peroxide reactivity and subsequent uranyl dissolution is partly caused by a blockage of reactive UO_2_ sites by U(vi) due to a low fraction of free carbonate species (HCO_3_^−^ + CO_3_^2−^). This could potentially suppress dissolution and the regeneration of the reactive sites. The fraction of free carbonate is expected to decrease with carbonate concentration due to the lower excess of total carbonate relative to U(vi) coordination centers (see Fig. 3b). This fraction is also expected to change slightly as time progresses due to changes in U(vi) and peroxide concentration. Even in 1 mM bicarbonate, more than half of the total carbonate (>0.5 mM) is expected to remain as free carbonate species. Prevented dissolution of U(vi) due to limitations in free carbonate species is thus not a likely explanation for the observed suppression of peroxide reactivity and subsequent uranyl dissolution.

As we lack the thermodynamic data to simulate the surface speciation, another possibility to consider is that the strong affinity of U(vi) towards O_2_^2−^ ligands could result in the formation of adsorbed uranyl-peroxo/uranyl-peroxo-carbonato complexes with low solubility in favor of readily soluble uranyl-carbonato complexes at the reactive sites. In this case, the addition of 40 mM would favor formation of soluble uranyl-carbonato complexes and increase the surface reactivity by removing the uranyl, thus removing the blockage of the reactive sites. However, as the passivating effect of uranyl on the peroxide reactivity towards the metal oxide surface has been observed for H_2_O_2_ degradation on ZrO_2_ (where no oxidized sites are formed)^10^ this is likely not the main reason for the observed peroxide passivation towards UO_2_.

To further explore the systems with high fractions of complexed peroxide, experiments with initial additions of 0.3 mM U(vi) in 1 and 10 mM bicarbonate were performed at different ionic strengths controlled by addition of NaClO_4_. The ionic strength was varied to explore the influence of electrostatic repulsion on the surface reactivity of complexed peroxide. Peroxide and U(vi) concentrations as functions of exposure time in 10 mM bicarbonate with an initial U(vi) concentration of 0.3 mM are presented in Fig. 6a and b, respectively.

**Fig. 5:**
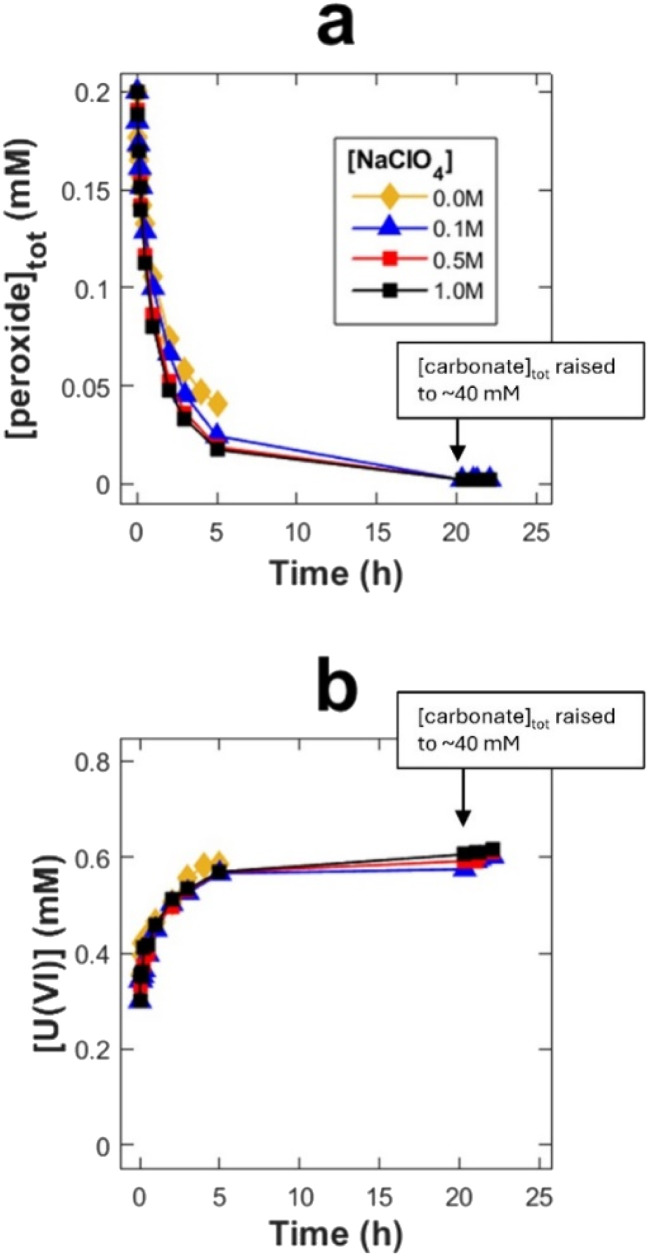
Peroxide concentration (a) and U(vi) concentration (b) as functions of exposure time in 20 ml solutions containing 10 mM bicarbonate, 0.3 mM initial U(vi) and 0–1 M NaClO_4_, with 30 mg UO_2_ powder suspension (SA/V = 8100 m^−1^).

In 10 mM carbonate, the ionic strength does not appear to significantly affect the rate of peroxide consumption (Fig. 5a) or the subsequent U(vi) dissolution (Fig. 5b). The absence of an ionic strength effect contradicts the previous hypothesis, that suppression of the peroxide consumption rate in systems with a high fraction of complexed peroxide is caused by electrostatic repulsion.^10^ Instead, we conclude that the complexed peroxide is inherently more stable than free H_2_O_2_ and therefore less reactive toward UO_2_.

**Fig. 6:**
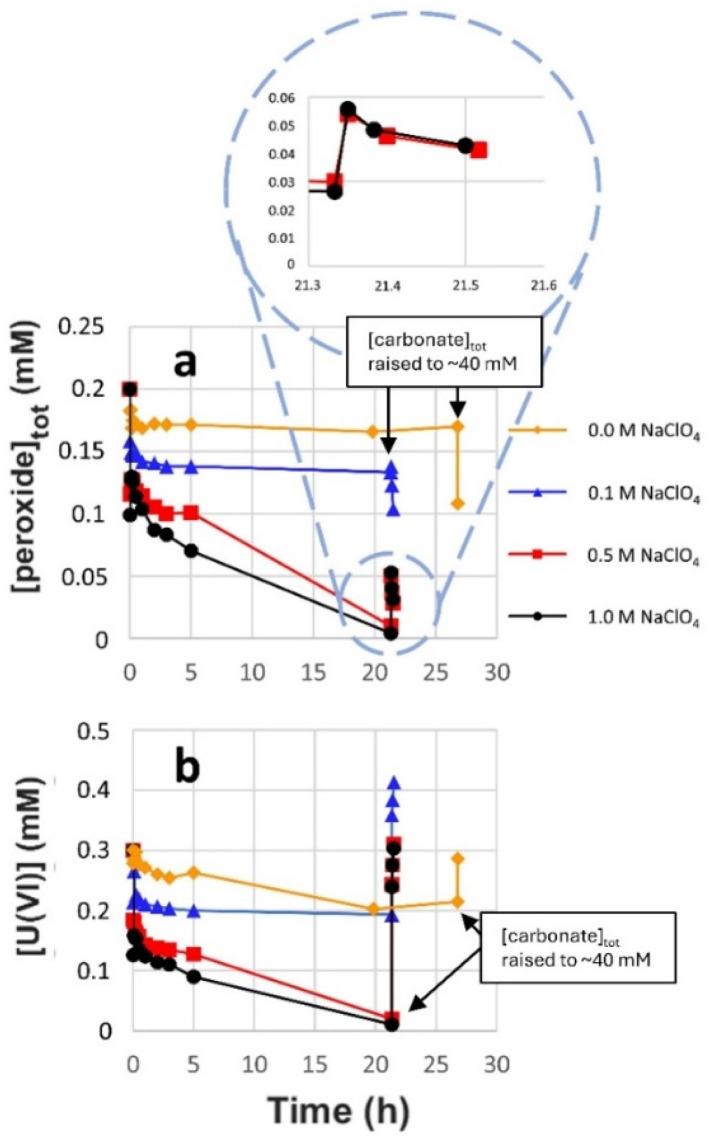
Peroxide concentration (a) and U(vi) concentration (b) as a function of exposure time in 20 ml solutions containing 1 mM bicarbonate, 0.3 mM initial U(vi) and 0–1 M NaClO_4_, with 30 mg UO_2_ powder suspension (SA/V = 8100 m^−1^).

Peroxide- and U(vi) concentrations as function of time for the corresponding exposures in 1 mM bicarbonate are presented in Fig. 6a and b, respectively.

Regardless of ionic strength, a fast initial drop in peroxide concentration (Fig. 6a) as well as in U(vi) concentration (Fig. 6b) can be observed. Increasing the ionic strength results in more substantial initial drops in the two concentrations. A similar effect has previously been observed in bicarbonate-free water.^15^ At the highest ionic strengths the U(vi) and peroxide concentrations keep decreasing after the initial fast drop and reach values close to the detection limit after 20 h. The apparent 1 : 1 ratio between the peroxide and uranyl disappearance from the aqueous phase in these two systems is likely caused by the formation of a secondary phase (such as studtite) on the UO_2_ surface. Upon increasing the bicarbonate concentration to 40 mM, a rapid increase in U(vi) concentration is observed at all ionic strengths. Interestingly, the peroxide concentration is also observed to initially increase with the bicarbonate addition at the two highest ionic strengths, followed by the type of decrease expected for reactive H_2_O_2_ (towards UO_2_). This initial, partial recovery of peroxide supports the idea of a peroxide decrease as a result of the formation of a secondary phase rather than a decrease caused by the typical H_2_O_2_ consumption on UO_2_ where the peroxide would be permanently lost.

At lower ionic strength where a significant amount of peroxide remains in solution, the peroxide concentration immediately starts to drop upon addition of bicarbonate. This indicates increased peroxide reactivity towards UO_2_ by the release of H_2_O_2_, as expected due to the formation of UO_2_(CO_3_)_3_^4−^ in favor of uranyl-peroxo-carbonato complexes (as was observed in NaClO_4_-free solutions (Fig. 2a)). The initial fast drop in U(vi) and peroxide concentrations in combination with the rapid increase in concentrations upon bicarbonate addition at the two highest ionic strengths, indicate that increasing the ionic strength favors adsorption of the uranyl peroxo-carbonato complex(es).

Calculated fractions of free H_2_O_2_ and free carbonate at the start of the exposures (*t* = 0) are presented in Fig. 5a and b respectively.

**Fig. 7:**
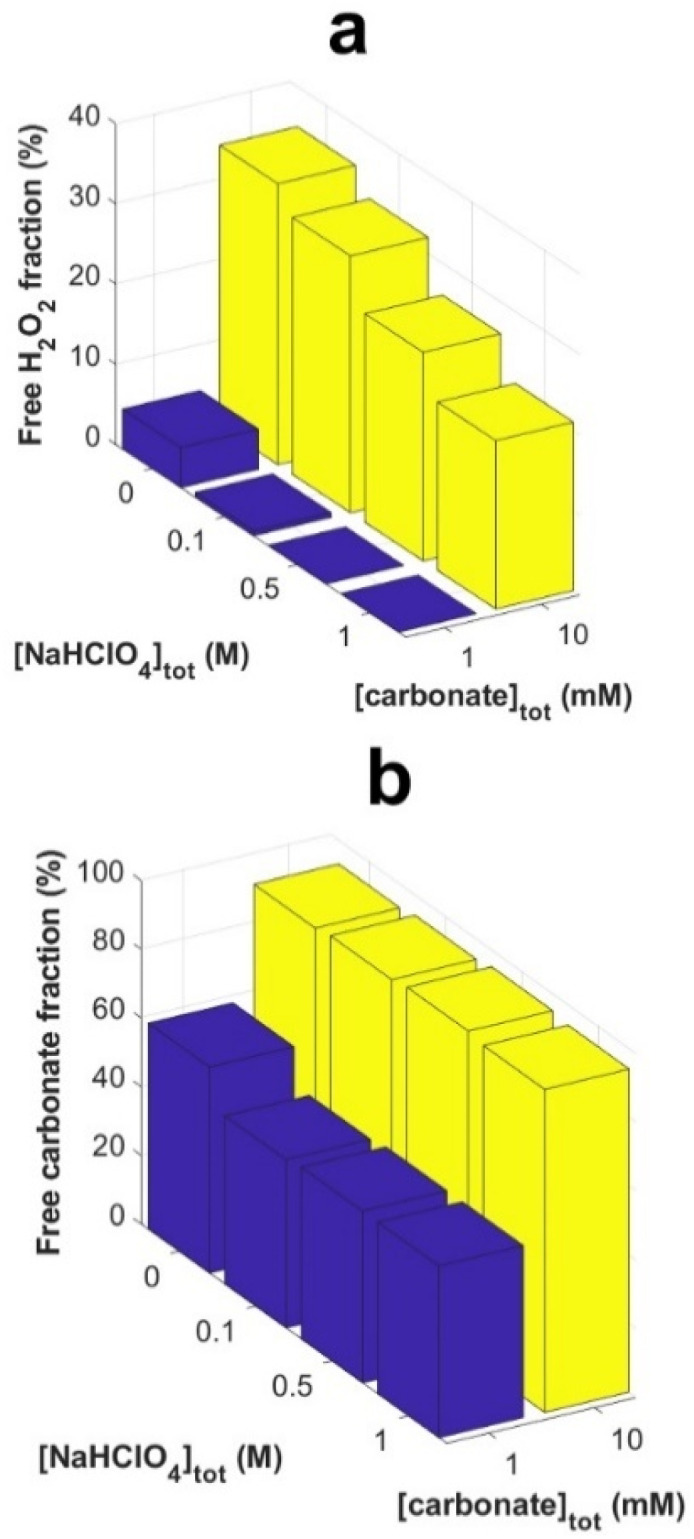
Calculated fractions (at *t* = 0) of free H_2_O_2_ (a) and free carbonate species (HCO_3_^−^ + CO_3_^2−^) (b) as functions of NaClO_4_- and total carbonate-concentration.

The fractions of free H_2_O_2_ (Fig. 7a) and free carbonate (Fig. 7b) change slightly with ionic strength but are mainly determined by the total carbonate concentration. At 10 mM bicarbonate, the fraction of free H_2_O_2_ is expected to decrease from 35 to 21 percent upon increasing the ionic strength. This is not reflected in the seemingly unaffected rates observed in Fig. 5a and b. Instead, the unaffected rates suggests that the stability of the coordinated O_2_^2−^ is not significantly affected by ionic strength (at least not at these concentrations of uranyl, peroxide and carbonate).

In the 1 mM bicarbonate system, the initial fraction of free H_2_O_2_ is expected to decrease from 5 percent to considerably less than 1 percent with increasing ionic strength. Hence, we would expect the consumption of peroxide and the release of U(vi) to be very slow and to become even slower with increasing ionic strength, provided that the screening of electrostatic repulsion between the uranyl-peroxo-carbonato complexes and the UO_2_ surface does not catalyze degradation of coordinated O_2_^2−^ on UO_2_. Any kinetic effect on the surface reaction between H_2_O_2_ and UO_2_ (if present) is masked by the continuous adsorption of uranyl and peroxide. However, the 1 : 1 ratio between loss of uranyl and peroxide in the aqueous phase is consistent with a low fraction of H_2_O_2_ to a point where loss of peroxide due to degradation of H_2_O_2_ on the UO_2_ is negligible.

## 4. Conclusions

The experiments performed in this work show that the bicarbonate concentration has little impact on the rate of H_2_O_2_ consumption on UO_2_ but a significant effect on the release of U(vi) in systems initially free from dissolved U(vi).

In systems initially containing U(vi), the concentration of bicarbonate has a significant effect on both the consumption of peroxide and the release of U(vi), likely by influencing the ratio between reactive, free H_2_O_2_, and non-reactive U(vi)-coordinated O_2_^2−^.

In 10 mM bicarbonate the consumption of peroxide as well as the release of U(vi) are unaffected by changes in the ionic strength while at lower bicarbonate concentration (1 mM), ionic strength is observed to influence the solubility of uranyl-peroxo-carbonato complexes. The combination of high ionic strength and peroxide predominantly in the form of U(vi)-coordinated O_2_^2−^ appear to favor adsorption of uranyl-peroxo-/uranyl-peroxo-carbonato species in bicarbonate deficient systems.

Speciation calculations based on the stability constants for uranyl-carbonato and uranyl-peroxo-carbonato complexes shed some light on the experimental observations. A stronger suppression of the peroxide reactivity towards UO_2_ is expected due to a lower fraction of uranyl-carbonato complex favoring higher fractions of passive peroxide in uranyl-peroxo-carbonate complexes. The absence of an ionic strength effect at high bicarbonate concentration shows that the reason for the low surface reactivity of the ternary peroxo complexes is not electrostatic repulsion, but likely due to an inherently higher thermodynamic stability compared to that of free H_2_O_2_.

## Conflicts of interest

There are no conflicts to declare.

## Supplementary Material

RA-014-D4RA02281E-s001
