# Correction: The influence of bicarbonate concentration and ionic strength on peroxide speciation and overall reactivity towards UO_2_

**DOI:** 10.1039/d4ra90065k

**Published:** 2024-06-05

**Authors:** Daniel Olsson, Hazal Aydogan, Mats Jonsson

**Affiliations:** a Department of Chemistry, KTH Royal Institute of Technology Stockholm SE-100 44 Sweden daniols@kth.se

## Abstract

Correction for ‘The influence of bicarbonate concentration and ionic strength on peroxide speciation and overall reactivity towards UO_2_’ by Daniel Olsson *et al.*, *RSC Adv.*, 2024, **14**, 16248–16254, DOI: https://doi.org/10.1039/D4RA02281E.

The authors regret the omission of a funding acknowledgement in the original article. This acknowledgement is given here.

The Royal Society of Chemistry apologises for these errors and any consequent inconvenience to authors and readers.

## Supplementary Material

