# Supplementary material for: The influence of bicarbonate concentration and ionic strength on peroxide speciation and overall reactivity towards UO2
Source: RSC Adv. 2024 May 20;14(23):16248–54. doi: 10.1039/d4ra02281e (PMC11104010; doi:10.1039/d4ra02281e)
Supplement: RA-014-D4RA02281E-s001 [file RA-014-D4RA02281E-s001.pdf]

Electronic Supplementary Material (ESI) for RSC Advances.
This journal is © The Royal Society of Chemistry 2024

Supporting Information to manuscript:
The influence of bicarbonate concentration and ionic strength on peroxide speciation and overall reactivity towards UO_2

Measured pH (used in speciation calculations). Exposures in; uranyl free bicarbonate solutions (Table S1), bicarbonate solutions with 0.3 mM added uranyl nitrate (Table S2) and bicarbonate solutions with 0.3 mM uranyl nitrate and varied concentrations of sodium perchlorate (Table S3).

Table S1. Measured pH in solutions without added uranyl

[Carbonate]tot (mM)	Time (min)	Measured pH
10	5	8.9
	100	9.3
	356	9.6
	420	9.8
5	1	8.5
	14	8.8
	180	9.5
2	1	8.3
	20	9.0
	38	9.1
	90	9.2
	145	9.3
	350	9.5
	917,	9.6
	1426	9.7
1	30	7.8
	85	8.6
	150	8.3
	330	9.0
	435	9.5
	1320	9.5
	1440	9.7
	1620	9.8

Table S2. Measured pH in solutions with 0.3 mM added uranyl

[Carbonate]tot (mM)	Time (min)	Measured pH
10	1	7.8
	4	8.5
	57	8.7
	325	9.2
	1210	9.8
5	1	7.2
	14	8.2
	47	9.2

	240	9.6
	1200	9.8
2	18	8.1
	35	8.3
	90	9.1
	145	9.2
	325	9.2
	929	9.3
	1425	9.4
1	1	6.9
	33	7.8
	85	8.0
	330	8.2
	435	8.9
	1440	9.3

Table S3. Measured pH in solutions with 0.3 mM added uranyl and varied ionic strength

[Carbonate]tot (mM)	[NaClO4]/ M	Measured initial pH (measured within 1 minute of exposure)	Measured final pH (prior to raising the carbonate concentration)
10	0.1	7.8	10.0
	0.5	8.2	9.9
	1	8.3	9.9
1	0.1	7.4	9.2
	0.5	7.6	9.8
	1	7.5	9.8
